# Inter‐ethnic differences in pharmacokinetics—is there more that unites than divides?

**DOI:** 10.1002/prp2.890

**Published:** 2021-11-02

**Authors:** Olusola Olafuyi, Nikita Parekh, Jacob Wright, Jennifer Koenig

**Affiliations:** ^1^ Division of Physiology, Pharmacology and Neurosciences School of Life Sciences University of Nottingham Nottingham UK; ^2^ Department of Pharmacology and Therapeutics King’s College London London UK; ^3^ Centre for Bioscience Education King’s College London London UK; ^4^ Division of Medical Sciences & Graduate Entry Medicine School of Medicine University of Nottingham Nottingham UK

**Keywords:** environmental factors, genetic polymorphisms, inter‐ethnic differences, pharmacokinetic differences

## Abstract

Inter‐ethnic variability in pharmacokinetics (PK) has been attributed to several factors ranging from genetic to environmental. It is not clear how current teaching in higher education (HE) reflects what published literature suggests on this subject. This study aims to gain insights into current knowledge about inter‐ethnic differences in PK based on reports from published literature and current teaching practices in HE. A systematic literature search was conducted on PubMed and Scopus to identify suitable literature to be reviewed. Insights into inter‐ethnic differences in PK teaching among educators in HE and industry were determined using a questionnaire. Thirty‐one percent of the studies reviewed reported inter‐ethnic differences in PK, of these, 37% of authors suggested genetic polymorphism as possible explanation for the inter‐ethnic differences observed. Other factors authors proposed included diet and weight differences between ethnicities. Most respondents (80%) who taught inter‐ethnic difference in PK attributed inter‐ethnic differences to genetic polymorphism. While genetic polymorphism is one source of variability in PK, the teaching of genetic polymorphism is better associated with interindividual variabilities rather than inter‐ethnic differences in PK as there are no genes with PK implications specific to any one ethnic group. Nongenetic factors such as diet, weight, and environmental factors, should be highlighted as potential sources of interindividual variation in the PK of drugs.

## Graphical abstract

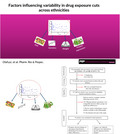

**Abbreviations**
AAG: acidic glycoprotein
CYP: cytochrome P450
HE: higher education
HIV: human immunodeficiency virus
PK: pharmacokinetics

## 1 INTRODUCTION

Understanding how the body handles drugs enables the design and optimization of dosing regimens that will achieve therapeutic plasma concentrations and deliver the desired therapeutic effects while avoiding subtherapeutic or toxic concentrations. It is common to observe differing therapeutic responses following drug administration; these differences are often attributed to interindividual differences in drug absorption, distribution, metabolism, or excretion.^1^ Interindividual differences in pharmacokinetic parameters have been linked to race, ethnicity, and ancestry, and prescribing recommendations have been directed at certain races or ethnicities.^1^

The terms race and ethnicity are often used interchangeably, and their meanings are not always clear despite attempts at standardization.^2^, ^3^ Ethnicity is often used to describe cultural and social factors^2^ and can have varying meanings across the English‐speaking world^3^ with some emphasizing the cultural aspects while others incorporate aspects of ancestry and race. The categories used vary between countries and the UN Statistics Division has noted that no internationally accepted criteria for ethnicity are possible.^4^ In human genetics, anthropology, and population genetics it is now accepted that ideas of race or ethnicity have no biological or genetic basis.^3^

Environmental factors, such as diet which has cultural and ethnic differences, have been shown to affect the pharmacokinetics (PK) of drugs.^5^ For example, many traditional Asian and African diets contain spices, vegetables, and herbs^6^ while a typical European diet is more likely to contain high levels of fat,^7^ though a recent study argued that the levels of fat in traditional Asian diet may be comparable to fat levels in Western diet.^8^ Some individuals may maintain their traditional diet irrespective of their geographical location. A study showed that, in subjects who consumed low‐fat vegetarian diet, there was a resultant slower absorption rate of paracetamol compared to their nonvegetarian counterparts. The authors explained that the difference may be due to reduced gastric emptying resulting from consumption of vegetarian diet.^9^ Green tea and its extracts, popular in East Asia but consumed throughout the world, are common and regularly used beverages and supplements; however, the polyphenols in the tea may affect absorption and metabolism by altering the levels of drug metabolizing enzymes and transporters involved in the drug disposition.^10^

Herbal medication, which is often used traditionally in some parts of the world, may have a broad range of effects on the PK of conventional medicines. In Western societies, St John's Wort is well known for its ability to interact with prescription medicines and there are numerous examples from other cultures.^11^ In South America, Cat Claw, a medicinal plant used by the Amazonians for its immunostimulant and antiviral properties, may inhibit the CYP3A4 isozyme,^12^ decreasing drug metabolism which therefore increases the plasma exposure of antiretroviral agents such as atazanavir, ritonavir, and saquinavir.^11^ Furthermore, garlic may selectively inhibit certain isozymes of cytochrome P450 (CYP) enzymes and induce expression of intestinal P‐glycoproteins thereby potentially influencing the absorption and elimination of drugs.^11^

Interindividual variations in drug distribution can also be attributed to variations in body weight or composition. Hayes et al., (2015) demonstrated that the weight‐for‐age in adults from different malaria endemic countries differ significantly ranging from an average of 50 kg in the South Asians to 68 kg in the Americas;^13^ this contrasts with the generally accepted average adult 72 kg weight based on healthy Caucasians.^14^ This weight variation may result in differences in the distribution kinetics of lipophilic drugs across these populations since higher body weight may be indicative of a higher fat mass and higher volume of distribution of such drugs.^15^

Plasma protein binding may influence the extent of drug distribution. The two main plasma proteins responsible for the binding of drug molecules in humans are α_1_ acidic glycoprotein (AAG) and the human serum albumin. Differences in α_1_ acidic glycoprotein (AAG) expression levels have been demonstrated between Whites and Chinese.^16^ Zhou et al., (1990) showed that there was a higher unbound fraction of disopyramide, propranolol, and diphenhydramine in Chinese patients (*n* = 8) compared to White patients (*n* = 8) and they attributed this to lower levels of AAG in the Chinese.^16^ Levels of AAG may be affected by a number of environmental factors^17^ and there are polymorphisms that show differences in AAG allele frequencies between ethnic groups.^18^, ^19^

Genetic polymorphisms of metabolizing enzymes and transporter proteins may affect several pharmacokinetic processes.^1^ One of the earliest reported and best‐known examples of this is the ethnic differences in response to alcohol due to striking differences in the presence of certain alleles for alcohol dehydrogenase and aldehyde dehydrogenase in European, Asian, Sub‐Saharan African, and African American groups.^20^ Polymorphisms have been widely reported in the CYP450 enzyme superfamily, a group of enzymes which are responsible for the metabolism of a wide range of drugs. For example, the functionally deficient allele, CYP2B6*6, prevalent in African Americans (46.7%) compared with Asians (17.4%) has been reported to be responsible for inter‐ethnic difference in the clearance of efavirenz. A review highlighted the clinical relevance of this observation in Asians and reported that due to the higher efavirenz plasma exposure observed in them following standard doses, optimization of dosing regimen may be warranted.^21^

There is substantial literature describing environmental and physiological differences that exist between ethnic groups some of which have pharmacokinetic significance and may result in inter‐ethnic variabilities in PK. Therefore, this study aims to explore literature evidence to determine the extent to which there are PK differences between ethnic groups, determine the mechanisms suggested for differences in PK in published literature, investigate how current teaching in higher education (HE) reflects the findings from literature, and recommend ways to assist educators in HE to approach the subject of inter‐ethnic differences in PK so that learners have a better understanding of the potential reasons why inter‐ethnic variation in PK may occur and potential impact these differences may have on treatment outcomes.

## 2 MATERIALS AND METHODS

### 2.1 Search strategy

To survey published literature that reported significant inter‐ethnic differences in the pharmacokinetic of drugs, a scoping review of literature was conducted. This involved a systematic approach whereby the preferred reporting items for systematic reviews and meta‐analyses (PRISMA) strategy was adapted to suit the purpose of this review and the process was conducted by two members of the project team such that each stage of the process completed by one member was checked separately by the other member of the team. When there was disagreement between the two members, the criteria used at the respective stage were reviewed by both members in order to maintain consistency. In this current study, while reviewing the literature on ethnic diversity in PK, terminologies similar to those used in published literature sources such as “Caucasians” for example, were used during literature search.

### 2.2 Initial search

To identify potentially relevant publications, two databases were used namely PubMed and SCOPUS, and the search term used for both databases is reported in Table S1 in the Supplementary Material. The selection of search terms used in identifying articles included terms such as “ethnic,” “blacks,” “African American,” “Asian,” “Caucasian,” and “European.” These terms were chosen because they are terms commonly attributed to race or ethnicity.

### 2.3 Screening of initial search

Duplicates were removed from the articles identified in the initial search of the two databases. The titles and abstracts from the identified articles were screened to remove those where inter‐ethnic differences were not mentioned in the context of differences in diet/lifestyle or absorption, distribution, metabolism, and excretion. Also, articles that did not involve humans were excluded.

### 2.4 Eligibility

After screening, the remaining articles were assessed based on whether they demonstrated differences in PK between ethnic groups. The articles which showed inter‐ethnic differences in PK were separated from those that did not show inter‐ethnic differences. Also, reviews and articles deemed not relevant to the research or already had the primary papers in the search were removed. The articles were deemed irrelevant if the authors did not directly measure pharmacokinetic parameters of drugs referenced in their study.

### 2.5 Included articles

The articles included in the final analyses excluded articles which did not have a pharmacokinetic implication for the differences observed.

### 2.6 Survey of teaching practices

A questionnaire that targeted academics who have either taught PK or are currently teaching PK in HE or the industry was designed and shared with contact of the project team through university email contact lists, social media platforms, and the British Pharmacological Society community forum. Responses were collected anonymously; no identifying information was required, and the questionnaire took less than 5 min to complete. The details of the questions asked in the survey are reported in Figure S1 of Supplementary Material 1 and Table S2 of Supplementary Material 2.

### 2.7 Analyses of articles and questionnaires included in the analysis

#### 2.7.1 Ethnic differences in PK in current teaching and the published literature

Analyses of the articles which showed inter‐ethnic difference in PK involved tallying the number of articles where authors reported inter‐ethnic differences in PK parameters that may be due to inter‐ethnic differences in one or more of the ADME processes. The percentage ADME tallies were determined to compare the number of articles where each ADME process was involved in inter‐ethnic variabilities to the total number of articles included in the analysis. In the questionnaire analysis, the number of respondents who thought there were inter‐ethnic differences in either one or more of the ADME processes was tallied. The percentage of those who thought any of the ADME process was involved in inter‐ethnic difference was determined in comparison with the total number of respondents. The percentages obtained from the article analyses and questionnaires were weighed equally and the resultant percentages were plotted on a bar chart.

The drugs and disease class of the drugs that were studied in the final included articles were tabulated; the table included the drug class, the key PK parameter differences between ethnic groups and the ADME processes that may have been affected, the authors’ explanation for inter‐ethnic difference observed, and the authors’ recommendation or other relevant remarks.

#### 2.7.2 Published literatures finding difference or similarities in PK between ethnicities

A comparison between studies that showed differences in PK and those that did not show differences in PK was conducted and presented as a percentage of the total number of PK publications in each decade of publication starting from papers published in 1980 till March 2021.

#### 2.7.3 Authors explanation/suggestions for inter‐ethnic differences in PK between ethnicities

The papers which reported inter‐ethnic differences in PK were screened to determine authors’ explanations or suggested reasons for inter‐ethnic differences in PK. The authors’ reasons were sorted in similar categories and represented as percentages of the total number of papers.

## 3 RESULTS

### 3.1 Literature search, screening, eligibility, and inclusion

**FIGURE 1:**
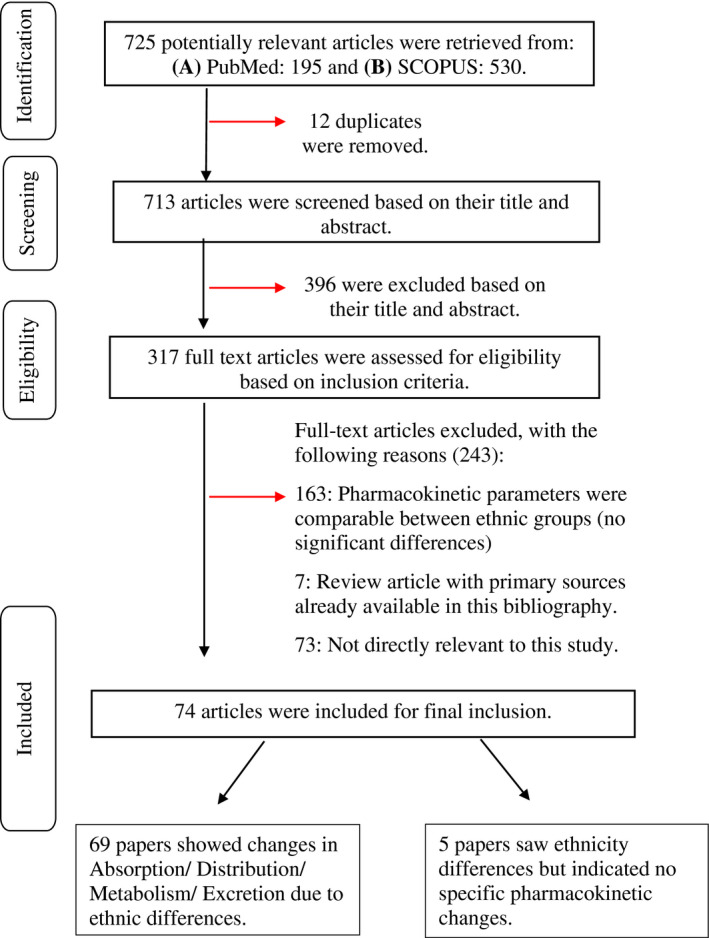
Search strategy implemented on PubMed and SCOPUS

A total of 69 studies were included in the final analyses. This was down from 725 studies identified from the initial literature search (Figure 1).

### 3.2 Summary of literature evidence showing inter‐ethnic differences seen across a wide range of drugs treatments.

From the literature, the most reported drug classes where ethnic differences were observed were those that treat cardiovascular diseases, central nervous system‐related conditions, and human immunodeficiency virus (HIV) (Table 1). Moreover, the most reported pharmacokinetic process affected for these drugs was metabolism resulting from polymorphisms of CYP enzymes and transporters. Only two papers reported excretion to be the pharmacokinetic process affected and both the drugs studied, phenytoin and morphine are used for the treatment of central nervous system disorders. Propranolol, nifedipine, paracetamol, rosuvastatin, midazolam, efavirenz, and cyclosporine were the most studied across multiple different papers in various ethnic groups.

**TABLE 1:** A summary of the inter‐ethnic differences in pharmacokinetics reported in the papers identified this current study

Drug class	Drug (Authors)	Authors’ terms for ethnicity groups studied	Result summary	Author's explanation of mechanism for inter‐ethnic differences	Authors remarks^e^	Pharmacokinetic processes involved
Cardiovascular	Statins^50^	Asians and Caucasians	Elevated expression of hepatic OATP1B1 and OATP1B3 in Asians compared to Caucasians.	The elevated expression is unexpected, suggesting that hepatic OATP expression alone does not explain the increased systemic statin levels in Asians compared with Caucasians.		Absorption^d^
	Pravastatin^51^	European Americans and African Americans	European Americans had a 41% higher AUC and 43% higher *C*_max_ than African Americans. Even after adjusting for genotype (SLCO1B1 521TC associated with significantly higher AUC and *C*_max_ and more prevalent in European Americans), gender, body surface area, and assay sensitivity. Oral clearance (Cl/F) was 1.4 times higher in African Americans than in European Americans	Ethnic differences in polymorphisms in transporter genes for the cell surface receptor OATP1B1 on hepatocytes which could affect the access of these drugs to the metabolizing enzymes in hepatocytes and hence the metabolic elimination of the drug.^b^		Absorption, distribution, and metabolism^d^
	Debrisoquine^39^	Japanese, Chinese, Egyptians, Saudi Arabians, Hong Kong, Caucasians, Nigerians, and Ghanaians	CYP450‐dependant oxidation polymorphisms may result in a poor metabolizer phenotype. The frequency of this phenotype for metabolism of debrisoquine is: 0%–0.5% in the Japanese, 0%–0.7% in the Chinese, 1%–1.4% in the Egyptians and Saudi Arabians, 2% in Hong Kong populations, and 6%–10% in Caucasians, Nigerians, and Ghanaians.		May affect the responses of the drug thus significant differences in dosage requirements between ethnic groups.	Metabolism^c^
	Propranolol^24^	Black and White subjects	53%–76% higher clearance in Black subjects	This is thought to be due to higher hepatic metabolism and increased hepatic blood flow in Black subjects.^b^		Metabolism^d^
	Propranolol^16^	Chinese and Caucasians	Plasma AGP concentration in healthy Chinese was lower than in Caucasians, whereas albumin levels were similar between the two groups. Consequently, unbound fractions of propranolol that reversibly interact with AGP are higher in the Chinese than in Caucasians.	Binding to plasma proteins reduces the extravascular distribution^a^		Distribution^c^
	Propranolol^39^	Caucasians and Chinese	AUC was 1.8 times higher in Caucasians than in the Chinese. Clearance was 1.7 times higher in the Chinese than in Caucasians	The Caucasians had a higher alpha‐1 acid glycoprotein level which is a major determinant of plasma protein binding of propranolol. A greater percentage of unbound propranolol would be expected to result in higher clearance.^b^		Distribution^d^
	Rosuvastatin^52^	East Asian (Chinese, Filipino, Korean, Vietnamese, and Japanese) and Caucasians	AUC _(0‐_ *_t_*_)_ was 1.7‐fold higher in the East Asian group compared to the Caucasians. East Asians had a maximum drug concentration that was 70%–98% higher than Caucasians.	The East Asian sub‐populations in this study reported lower daily cholesterol intake than the Caucasian population. Moreover, the Japanese reported a significant lower daily caloric intake while both the Japanese and Vietnamese had a lower percentage saturated fat intake.^a^ It was not clear whether this observation accounted entirely, if at all, for the observed differences in AUC and *C*_max_ ^b^		Absorption^d^
	Rosuvastatin ^53^	Chinese, Japanese, and Caucasian	AUC was 86% higher in the Chinese and 55% higher in the Japanese compared to Caucasians.	Polymorphisms in the transporters SLCO1B1 or ABCG2 may play a role.^b^		Absorption^d^
	Rosuvastatin^54^	Asians and Caucasians	Cl/F was reduced by 43.7% in Asians compared to Caucasians. Therefore, the Asian plasma exposure levels were around two times greater.			Not Specified
	Losartan and its active metabolite E‐3174^55^	Koreans, Hans, Mongolians, Hui, and Uighurs	Losartan: Clearance was significantly higher in Hans compared to the Mongolians (41%) and Hui (36%). E‐3174: *t*_1/2_ was significantly lower in Koreans (33%), Hui (27%), and Uighurs (37%) compared to Hans. *t*_1/2_ was significantly lower in Koreans (30%), Hui (23%), and Uighurs (33%) compared to Mongolians. *V*_d_/weight was significantly lower in Koreans (44%), Hui (43%), and Uighurs (38%) compared to Hans *V*_d_/weight was significantly lower in Koreans (33%), Hui (32%), and Uighurs (26%) compared to Mongolians. *C*_max_ was significantly higher in Koreans (51%), Hui (48%), and Uighurs (49%) compared to Hans *C*_max_ was significantly higher in Koreans (27%), Hui (23%), and Uighurs (25%) compared to Mongolians.	The authors suggest that further investigation into polymorphisms of CYP2C9s’ influence into these differences is warranted.^b^		Distribution^d^
	Felodipine^27^	Caucasians and Mexicans	Bioavailability of felodipine was around 1.5 times higher in Mexicans compared to Caucasians.	Thought to be due to decreased CYP3A4 enzymatic activity in Mexicans indicated by previous investigations.^b^		Metabolism^d^
	Atorvastatin^53^	Chinese, Japanese, and Caucasian	AUC was 53% higher in the Chinese and 69% higher in the Japanese compared to Caucasians.	Polymorphisms in the genes encoding transporters SLCO1B1 or ABCG2 may play a role.^b^		Absorption^d^
	Simvastatin^53^	Chinese, Japanese, and Caucasian	AUC was 23% higher in the Chinese and 12% higher in the Japanese compared to Caucasians.	Polymorphisms in the genes encoding transporters SLCO1B1 or ABCG2 may play a role.^b^		Absorption^d^
	Levosimendan^26^	Chinese and Caucasians	Clearance was 1.7‐fold lower in the Chinese and elimination half‐life was longer by 1.6‐fold in the Chinese compared to Caucasians.	This may be explained by higher protein binding rates as the percentage of protein binding of plasma levosimendan being 99.15% in the Chinese subjects.^b^		Distribution^d^
	Warfarin^56^	Asians, White, and African Americans	The mean maintenance dose was 6.1 mg in African Americans, 5.1 mg in Whites, and 3.4 in Asian Americans, that is, warfarin requirements were highest in African Americans, intermediate in Whites, and lowest in Asians. Polymorphisms in the promoter region on CYP2C9 are associated in Asians and suggest a lower dose requirement		It is recommended that Asians should be started with an approximately 50% lower dose than African Americans and Whites	Distribution^d^
	Nifedipine^57^	South Asian and Caucasian	South Asians have 1.7‐fold higher AUC compared to Caucasians. However, bioavailability remained similar between the two groups suggesting systemic clearance to be the cause. Systemic clearance was 50% lower in south Asians compared to Caucasians.	This may be due to genetically determined lower hepatic CYP3A4 activity in south Asians.^b^		Metabolism^d^
	Nifedipine^58^	Taiwanese, British and Americans.	69.7% of Taiwanese subjects can be classified as slow metabolizers. *C*_max_ and AUC were higher, across several studies, in the Taiwanese compared to British and American subjects.	Postulated this occurs due to ethnic differences in CYP3A activity^b^		Metabolism^d^
	Nifedipine^59^	South Asian and Caucasian	AUC of nifedipine was threefold higher in South Asians than in Caucasians. The AUC of nifedipine and AUC of its first pass metabolite, nitropyridine, ratio was higher in South Asians (4.6 ng ml^−1^ h) compared to Caucasians (2.3 ng ml^−1^ h). *t*_1/2_ was 5.5 h lower in Caucasians	.	Lower doses recommended for therapeutic value in South Asians	Metabolism^d^
Central Nervous System‐related drugs	Modafinil^31^	Koreans, Uighurs, and Hans	Clearance was higher in Koreans (25%) and Uighurs & Hui (12%) compared to the Hans.	It is suggested that diet may play a role in this as all the groups in this study are geographically close together but vary in diet.^b^		Metabolism^d^
	Haloperidol^60^	Chinese and non‐ Chinese (Blacks, Hispanics, and Caucasians).	Haloperidol plasma concentration was 40%–50% higher in the Chinese subject compared to the non‐Chinese Ethnicity was a significant factor for Blacks and Chinese. Haloperidol doses for comparable plasma levels required for Caucasians and Black groups were significantly greater than the Chinese.		Lower dosing for Chinese is required.	Metabolism^d^
	Clozapine^61^	Caucasians and Asians	Mean dose of clozapine was 2.5 times higher in Caucasians compared to Asians to achieve similar therapeutic effects however there were no significant differences in mean plasma clozapine levels. This further suggests that there are ethnic differences	It was postulated that this may be due to reduced CYP1A2 activity seen in Asians.^b^ Another possibility may be due to polymorphisms of the CYP1A2 gene which in smokers of Caucasian backgrounds, has been inducible hence requiring the higher doses of clozapine.^b^		Metabolism^d^
	Ziprasidone^32^	Mongolians and Hans	The dose required to achieve therapeutic plasma concentration was lower in the Hans than the Mongolians. The concentration to dose ratio was higher for the Hans than the Mongolians This maybe because the Hans have slower metabolism.	The authors postulated that genetic factors may have some attribution for these differences however provide no evidence. Moreover, diet may play an important role as there was a marked difference between the Hans, whose diet is more agriculturally based, and the Mongolians, whose diet is rich in fat and protein.^b^		Distribution^d^
	Bitopertin^62^	Caucasians, Chinese and Japanese	Clearance was 1.20‐fold higher in the Chinese compared to Caucasians. Clearance increased by 1.17‐fold in the Japanese compared to the Caucasians. This study also used physiologically based pharmacokinetics (PBPK) prediction modeling to assess ethnic sensitivity for a pathway. PBPK predicted a similar value of increased clearance of 1.32‐ and 1.27‐fold in the Chinese and Japanese, respectively, compared to Caucasians.			Distribution^d^
	Phenytoin^63^	Ghanaians and Caucasians	The excretion of PHP was 3–4 times greater in Caucasians than Ghanaians.			Excretion^c^
	Paracetamol (acetaminophen)^64^	South African, Whites, and Asians	Mean clearance was faster in South African villagers compared to Whites and Asian immigrants in London.	Subjects were in “near basal” condition. Possible reasons for this difference may be oral contraception, alcohol, tobacco, and environmental factors which may modify the rate of paracetamol conjugation.		Distribution and Metabolism^d^
	Paracetamol (acetaminophen)^33^	Subjects from Hong Kong Chinese, Pakistan, Denmark, Spain, and South Africa	In the Hong Kong Chinese subjects, half‐life was 15%–62% higher compared to subjects in Pakistan, Denmark, Spain, and South Africa and oral clearance was 16%–56% slower.	Maybe due to environmental factors such as social drugs (ethanol), hormonal contraceptives, and absence/presence of a meat diet. Further comparative studies are required.^b^		Distribution^d^
	Paracetamol (acetaminophen)^65^	Asians, Whites, Ghanaians, and Scottish	Mean paracetamol clearance was 21% lower in Asians than in Whites. The proportion of paracetamol excreted as mercapturic acid and cysteine conjugates was significantly lower in Ghanaians compared to Scottish subjects.	Ghanaians may be at less of a risk of hepatotoxicity from paracetamol as the oxidative pathway that produces the hepatotoxic metabolites are detoxified to mercapturic acid and cysteine conjugates (which were less in Ghanaians)^a^		Metabolism^c^
	Repinotan^66^	Caucasians and Japanese	Mean Clearance was about two times lower in Caucasians than the subjects of Japanese origins.	The authors postulated that may be due to ethnic differences in CYP2D6 activity.^b^		Metabolism^d^
	Adinazolam and N‐demethyladinazolam (NDMAD)^67^	Asians, African Americans, and Caucasians	For Adinazolam: Asians had higher *C*_max_ (27% and 29%) and AUC (26% and 38%) compared to African Americans and Caucasians, respectively. Asians had 1.3 times faster clearance than African Americans and 1.6 times faster clearance than Caucasians. For NDMAD: Asians had higher *C*_max_, 20% and 29%, than African Americans and Caucasians, respectively. Asians had higher AUC by 10% and 17% than African Americans and Caucasians, respectively.		African Americans seemed to display more of the adinazolam benzodiazepine effects of higher sedation and reduction of psychomotor performance than Caucasians which could be explained by more extensive metabolism into the active metabolite.	Metabolism^d^
	Codeine^68^	Chinese and Caucasians	Excretion of metabolized codeine, codeine‐6‐glucuronide, was higher in Caucasians (62%) compared to the Chinese (44%)	The Chinese are less able to metabolize codeine (by conjugation with glucuronic acid).^a^		Metabolism^c^
	Desipramine^69^	Chinese and Caucasians	Total plasma clearance was higher by 1.7‐fold in Caucasians than the Chinese.	The authors speculated that this may be due to genetic polymorphisms differences between the two groups involving hepatic metabolism relating to hydroxylation. However, environmental factors may also play a part.^b^		Metabolism^d^
	Midazolam^70^	Caucasians and Mexicans	Bioavailability, expressed as AUC, was about 4.5‐fold higher in Mexicans compared to Caucasians speculated to be due to decreased systemic clearance.	CYP3A4‐mediated biotransformation inter‐ethnic variation may be the cause of the higher bioavailability.^b^		Metabolism^d^
	Midazolam^25^	Han, Mongolian, Korean, Hui, and Uighur	*C*_max_ in Mongolians was significantly lower than Hans (1.4‐fold), Uighur (1.7‐fold), Huis (1.7‐fold), and Koreans (2.5‐fold). *T*_max_, in the Hans was 0.7 h longer than the Koreans and 0.9 h longer than Uighurs. The half‐life of midazolam in Koreans was 1.7 times higher than in Mongolians, 1.6 times higher in Hans compared to Uighurs, and Koreans had double the half‐life of midazolam than Uighurs.	Potential explanation for the differences seen in the Mongolians reported was that three of the participants in the Mongolian cohort did not sleep during the study period. Others in the Mongolian groups slept for shorter lengths compared to the other ethnic groups which may account for the lower *C*_max_ and half‐life.^a^		Not Specified
	Nicotine ^71^	European Americans and African Americans	European Americans had a 1.6‐fold more nicotine glucuronidation excretion than African Americans While under the nicotine patch, African Americans excreted less nicotine glucuronide conjugates compared to European Americans, 18.1% and 29.3%, respectively. While under the nicotine patch, African American excreted less cotinine glucuronide conjugates compared to European Americans, 41.4% and 61.7%, respectively.	This may be due to decreased N‐glucuronidation and decreased oxidation. Previous studies have shown that African Americans have 25% lower metabolic clearance of nicotine to cotinine.^b^		Metabolism^d^
	Morphine^72^	African Americans and Caucasians	Lower morphine clearance in Caucasians compared to African Americans which may be due to higher frequencies of defective OCT1 variants in Caucasians. Bodyweight, genetic variants coding for loss‐of‐function of OCT1 play significant roles in early morphine clearances			Excretion^d^
	Morphine^73^	Caucasians, native Indians, and Latinos	Caucasians had higher plasma levels of M6G than Indians (30%) or Latinos (24%).	The native Indians experienced more suppression of the ventilatory response to C0_2_. This may be due to Caucasians being more resistant to morphine effects or the metabolites of morphine.^b^	Despite this difference, Caucasians did not exhibit a higher incidence of adverse effects after morphine administration.	Metabolism^d^
	Tizanidine^74^	Caucasians and Japanese	Clearance was twofold higher in Caucasians than in Asians Metabolic clearance was 1.4‐fold higher in Caucasians than in the Japanese			Metabolism^d^
	Alprazolam ^75^	Caucasian, American‐born Asian, and Foreign‐born Asian	When body surface area was considered following oral administration of Alprazolam: AUC was 20% higher in foreign‐born Asians and 25% higher in American‐born Asians compared to Caucasians. Clearance was 36% slower in American‐born Asians and 28% slower in Foreign‐born Asians compared to Caucasians.	Both the Asians groups shared similar diets which may affect the pharmacokinetics similarly.^b^	Lower doses may be required for Asians to achieve similar steady‐state alprazolam blood concentrations	Absorption & Distribution^d^
	Diazepam^76^	Chinese and Caucasians	*T*_max_ was longer in the Chinese compared to the White Caucasians. Volume of distribution was 52% higher in White Caucasians.	As subjects were not fasting, differences in meals between the two groups could affect the rate of absorption thus a longer *T*_max_ ^b^ The White Caucasians were older, heavier, taller, and had a greater skin fold thickness which may correspond to slower distribution^a^		Absorption & Distribution^d^
Antiretroviral drugs	Efavirenz^36^	Chinese, Whites, and Blacks	The Chinese had a higher *C*_max_ than the Whites and Blacks by 1.4‐fold. The clearance was faster in Whites and Blacks by twofold compared to the Chinese	A potential reason for this may be that EFV plasma concentration variations maybe due to lower concentration of EFV in comparison to increasing body weight, as reported by a regression study. Another attributing factor may be due to genetic variations in CYP2B6.^b^		Distribution and metabolism^d^
	Efavirenz^77^	Ethiopians and Tanzanians	CYP2B6 polymorphisms allele frequency was significantly higher in Tanzanians (41%) relative to Ethiopians (31%) and ABCB1 polymorphisms are associated with higher plasma EFV plasma concentration. Even after controlling for these polymorphisms, EFV polymorphisms remains significantly higher in Tanzanians compared to Ethiopians.	CYP2B6 and ABCB1 genotype, interactions of genetics and the environment may affect the plasma levels of EFV therefore affecting the immunological response^b^		Metabolism^c^
	Efavirenz^21^	African Americans, Sub‐Saharan Africans, Hispanics, Caucasians, Japanese, and Asians.	The slow metabolizer of EFV CYP2B6516 G>T polymorphism is important for identifying characteristics for patients at a higher risk of elevated plasma concentration of EFV in different ethnic groups. This polymorphism was found to be significantly higher in African Americans (46.7%) and Sub‐Saharan Africans (45%) than in Hispanics (17.4%), Caucasians (21.4%), Japanese (18%), and Asians (17.4%).	Genotyping for CYP2B6 enzymes maybe useful for dose optimization		Metabolism^c^
	Efavirenz^78^	Hispanics and other (Hispanic, Asian, American Indian/Alaskan, and multiracial)	AUC was about 8% higher in White/non‐Hispanics than others.			Not Specified
	Lamivudine (3TC)^36^	Europeans and Chinese	Europeans had a 1.3‐fold higher *C*_max_ than the Chinese when given the once a day dose. When given the twice a day dose there was also a 1.6‐fold increase in *C*_max_ in the Europeans. Systemic exposure was higher in Europeans compared to the Chinese for both the once and twice a day dosing by 1.3‐fold and 1.4‐fold, respectively.	The authors attributed these observed differences between ethnicities to be potentially due to body weight, genetic polymorphisms, and concomitant medication.^b^		Not Specified
	Tenofovir disoproxil fumarate (TDF^36^)	African Americans, Ugandans, Thai, and Chinese	The systemic exposure (AUC) of TDF was higher in HIV‐infected Chinese (with combination therapy including TDF) by 2.9‐fold compared to HIV‐infected Ugandans (combination therapy including TDF) and by 1.6‐fold compared to HIV‐infected African Americans (combination therapy including TDF). The systemic exposure (AUC) of TDF was higher in HIV‐infected Thai (combination therapy including TDF) by 2.2‐fold compared to HIV‐infected Ugandans and by 1.2‐fold in HIV‐infected African Americans.	Maybe due to lower body weights in Asians^b^		Distribution^d^
	Nevirapine (NVP)^36^	Chinese, Ugandan, Indian, and South Africans	The Chinese had higher *C*_max_ than the Indians by 1.3‐fold, the Ugandans by 1.3‐fold, and the South Africans by 1.7‐fold The Chinese also had higher AUC than the Indians by 1.3‐fold, the Ugandans by 1.5‐fold, and the South Africans by 1.7‐fold.	Maybe due to lower body weights in Asians^b^		Not Specified
	Lopinavir and ritonavir (LPV/r)^36^	Chinese, Whites, Blacks, and Asians	*C*_max_ was 1.1‐fold higher in the Chinese compared to the other participants. AUC_0‐_ *_t_* was 1.6‐fold higher in the other groups when compared to the Chinese	Maybe due to lower body weights in Asians^b^		Distribution^d^
Immunosuppressant	Cyclosporine (CsA)^79^	Native Americans, Whites, Africans, Orientals, Latin Americans, and French	Oral clearance in Native Americans is one third the clearance seen in other ethnic groups (Whites, Africans, Orientals, Latin Americans, and French).	This may be due to polymorphisms in the CYP3A enzyme or P‐glycoproteins.^b^		Metabolism^d^
	Cyclosporine (CsA)^80^	North Americans and Mexicans	Mexicans displayed 1.7‐fold higher *C*_max_ and 1.5‐fold higher bioavailability in comparison to North American Caucasians	A possible explanation may be variation of CYP3A4 activity in the gut which is determined by either genes or nutrition consequently resulting in impaired first pass metabolism.^b^		Metabolism^d^
	Cyclosporine (CsA)^81^	African Americans and Caucasians	AUC was 1.2‐fold higher in African Americans in the first 2 months. Increased AUC suggests the bioavailability might be higher (although further studies would be required).	Ethnic disparity may be due to immunological hyper‐responsiveness and/or bioavailability of the immunosuppressants^b^		Absorption^d^
	Grape juice (GJ) effects of microemulsion cyclosporine (CsA)^82^	African Americans and Caucasians	GJ increased peak concentration of CsA in African Americans by 39% and in Caucasians by 8% GJ increased exposure of CsA by 60% in African Americans but only by 44% in Caucasians. Absolute bioavailability of CsA, when given water, was 22% lower in African Americans compared to Caucasians. However, there was no difference in absolute bioavailability between the two groups when CsA was given with GJ.	GJ increases bioavailability of CsA in African Americans much more than in Caucasians.^a^		Absorption^c^
	Mycophenolic Acid (MPA)^83^	Asians, Caucasians, and Africans	Asians have higher MPA exposure than Caucasians, with comparable doses, seen across multiple studies. Clearance was 1.8 times faster in Caucasians compared to Asians. Frequency of the UGT1A9*1 allele was 15% in Caucasians, 28% in Africans, and absent in Asians.		Carriers may require higher doses.	Metabolism^c^
	Mycophenolic Acid (MPA)^84^	African American males and Caucasians	MPA was cleared 1.5 times faster in African Americans compared to male Caucasians and 1.6 times faster in African Americans compared to Caucasian females. Appears to be related to less enterohepatic circulation in African Americans (23%) compared to both Caucasian males (42%) and females (50%)	It was postulated by the authors that these differences may be due to either SNPs in metabolism enzymes or efflux transporters in the hepatic or intestinal epithelium however no evidence was given.^b^		Metabolism^d^
	Tacrolimus (Mohamed ME. et al)	African American, European, Native Americans, and Asian ancestry	African American had the lowest dose‐normalized tacrolimus trough concentrations due to higher frequency of CYP3A5 expressers (CYP3A5*3, CYP3A5*6, and CYP3A5*7) compared to European, Native Americans, and Asian ancestry.			Metabolism^c^
	Tacrolimus^38^	African Americans, Latin Americans, and Whites	After oral administration: *C*_max_ was 1.8 times higher in Whites compared to the African Americans. Absolute bioavailability was significantly lower in African Americans (11.9%) and Latin Americans (14.4%) than in Whites (18.8%).	May be due to intestinal CYP3A or P‐glycoprotein activity differences. Evidence from this study highlights the importance of intestinal counter transport which regulates the exposure to drugs for metabolism.^b^		Absorption^d^
	Sirolimus^85^	Thai and Caucasians	The Caucasians had 1.5 times higher AUC than the Thai. The Thai had a 1.2‐fold rapid clearance and a lower absorption rate compared to Caucasians.	The difference of PK may be explained by P‐glycoprotein and CYP4503A4 enzyme inter‐ethnic differences.^b^		Absorption and Metabolism^d^
Antineoplastic drugs	Doxorubicin^86^	Chinese, Malays, and African Americans	Chinese (0.426) and Malays (0.514) had lower frequencies of the CBR3 11G allele compared to Indians (0.704). Chinese (0.426) had a lower frequency of the CBR3 11G allele than African Americans (0.727)	CBR3 11G allele is associated with metabolism of doxorubicin and with lower doxorubicin AUC/ doxorubicinol AUC metabolites ratio, lower CBR3 expression in breast tumor tissue and greater tumor reduction.^a^		Metabolism^c^
	Doxorubicin^87^	Caucasians, Chinese, and Indians	Patients that are homozygous for CBR3 11G>A allele are associated with lower AUC ratio of doxorubicin and its metabolite, doxorubicinol. Therefore, there is less accumulation of doxorubicin and associated toxicity. This allele is common in the Chinese compared to Indians and Caucasians.			Metabolism^c^
	Alisertib^88^	East Asians and Western subjects (2015 study)	67% higher dose‐normalized exposure (AUC_0‐_ *_t_*) of Alisertib in East Asians compared to Western subjects. 40% lower geometric mean apparent clearance in East Asians compared to Western subjects leading to a 70% higher mean dose‐normalized, steady‐state systemic exposure	Differences may be due to polymorphisms in enzymes involved in metabolism.^b^		Metabolism^d^
	Alisertib^34^	East Asians, West Asian, and Western subjects (2018 study)	52% higher relative bioavailability in East Asian subjects compared to Western subjects.			Absorption^d^
	Selumetinib^37^	Asians (Japanese, non‐ Japanese Asians, or Indians) and Western subjects (Blacks)	Selumetinib exposure was higher in all Asians compared to Western subjects when normalized by dose per kg of body weight. Dose‐normalized AUC was 35% higher in Asians compared to Western subjects. Dose‐normalized *C*_max_ was 39% higher in Asians compared to Western subjects	The study suggests that differences may not only be due to due to body weight differences only. Genetic variations may also potentially contribute.^b^		Distribution and Metabolism^d^
Urinary incontinence	Tolterodine (5‐hydroxymethyl (5‐HM) is the active metabolite)^89^	Koreans and Japanese	AUC for 5‐HM was 40% higher in Koreans compared to the Japanese. The proportion of variance due to ethnicity was 12.9%, as indicated by ANCOVA	Variations may be due to intrinsic clearances and genotype frequencies.^b^		Metabolism^d^
Anti‐diabetics	Repaglinide^90^	Japanese and Caucasians	Plasma concentration of repaglinide was about 1.2‐fold higher in the Japanese than the Caucasians.	Thought to be due to metabolism, particularly polymorphisms in the enzyme CYP3A4, resulting in higher plasma concentration in the Japanese.^b^		Metabolism^d^
Anti‐allergy	Diphenhydramine^91^	Oriental and Caucasian	*V*_d_ was 1.7‐fold higher in Orientals compared to Caucasians.	Unbound diphenhydramine in plasma was higher in Orientals (24%) than Caucasians (14.8%). This provides a potential explanation for the increased *V*_d_ in oriental subjects.^a^		Distribution^d^
Anti‐infective	Tinidazole^92^	Han, Mongolian, Korean, Hui, and Uighur	Half‐life was around 18% shorter in Uighurs compared to the other ethnicities. Systemic exposure AUC (0‐*t*) was lower in Uighurs compared to the other ethnicities. Clearance was 20% higher in Uighurs compared to the other ethnicities.	A potential reason for this, identified in the paper, was that the Mongolians, Hans. Koreans and Huis’ share similar Asian decent, whereas the Uighurs’ share characteristics with Caucasians. Tinidazole is primarily metabolized by the CYP3A4 isozyme. CYP3 activity has previously been shown to be decreased in Caucasians compared to Asians. This may account for the higher clearance and shorter half‐life.^b^		Metabolism^d^
	K‐601 (hospital prepared medicinal formulation in China) for influenza and cough treatment.^93^	Africans (Ghana, Zambia, and Nigeria)	The AUC for the African subjects was 4‐ to 10‐fold higher than the Chinese for three of the isolated compounds found in the formulation consisting of benzylisoquinoline alkaloids. *T*_max_ for the compounds was fourfold faster in the Chinese subjects compared to the Africans.	The authors postulated that (1) the compounds are poorly absorbed by the Chinese or they metabolize it too fast and (2) the African subjects may absorb the compounds slower or metabolize it slower than the Chinese subjects.^b^		Absorption^d^
NSAID	Acetyl salicylic acid (ASA)^94^	Otomies and Mesticians	Metabolism of ASA to all its metabolites (gentisic acid (GA), salicylic acid (SA), and salicyluric acid (SUA)) was significantly slower by 2.5‐fold in Otomies compared to Mesticians.			Metabolism^c^
	Meloxicam^95^	Germans and Mexicans	Clearance was about 1.2‐ to 1.6‐fold higher in Germans compared to Mexicans.	Polymorphisms of CYP3A4 may play a role as it has previously been shown that drugs metabolized by this enzyme, including meloxicam, is increased in Mexicans.		Metabolism^d^
Anti‐ulcer	Omeprazole^96^	Chinese and Whites	Omeprazole AUC was higher by 1.7‐fold in the Chinese compared to White subjects. Oral clearance was greater in Whites by 1.7‐fold compared to the Chinese.	Clearance of Omeprazole has been shown to be strongly correlated with polymorphic mephenytoin system, involving CYP2C19. Therefore, this difference may be due to higher proportion of heterozygous extensive metabolizers of mephenytoin in oriental subjects. Also, another possible reason may be due to increased bioavailability in the Chinese due to decreased first pass metabolism.^b^		Metabolism^d^
Menopause	Ibandronate^97^	Taiwanese and Caucasian	Taiwanese subjects displayed a 2.41‐fold greater AUC and 1.69‐fold greater *C*_max_ compared to Caucasians. Cl/F was 2.48‐fold lower on Taiwanese subjects compared to Caucasians.	As ibandronate is not bio‐transformed and largely excreted in the urine, the total body clearance will be close to renal clearance suggesting that the bioavailability is greater in Taiwanese. May explain the differences in Cl/F.^b^		Distribution and Metabolism^d^

Note

ABCG2, ATP‐binding cassette super‐family G member 2; AAG, α_1_ acidic glycoprotein; AUC, area under the curve; CBR, carbonyl reductase 1; Cl/F, Oral clearance; *C*_max_, maximum serum concentration that a drug achieves; CYP, cytochrome P450; CYP, cytochrome P450; M6G, Morphine‐6‐glucuronide; NSAID, non‐steroidal anti‐inflammatory drugs; OATP, organic anion transporting polypeptides; OCT1, organic cation transporter 1; SCLO1B1, solute carrier organic anion transporter family member 1B1; SNPs, single‐nucleotide polymorphisms; *t*_1/2_, half‐life; *T*_max_, time taken to reach *C*_max_; UGT1A9, UDP glucuronosyltransferase family 1 member A9; *V*_d_, Volume of distribution; *V*_d_, volume of distribution.

^a^ The authors explanation or mechanism for inter‐ethnic difference is evidence based on data provided in the respective paper.

^b^ The authors explanation or mechanisms for inter‐ethnic differences was postulated and no evidence was found in the respective paper.

^c^ Observed results and pharmacokinetic parameters to be evidence based.

^d^ Observed results and pharmacokinetic parameters to be speculative and no evidence was found to support this in the respective papers.

^e^ Authors remarks: Additional information, if provided, on inter‐ethnic differences or dose adjustment recommendations from the papers’ authors.

Additionally, from all the statins included in this review, systemic absorption was mainly affected between the studied ethnicities. Moreover, polymorphisms of genes encoding the transporters OATP1B1, ABCG2, and SLCO1B1 were linked to the ethnic differences.

Overall, those classified as Caucasians (sometimes referred to as White) and Asians were the two most commonly studied.

### 3.3 Papers showing ethnic differences in pharmacokinetics compared to those showing no significant differences.

**FIGURE 2:**
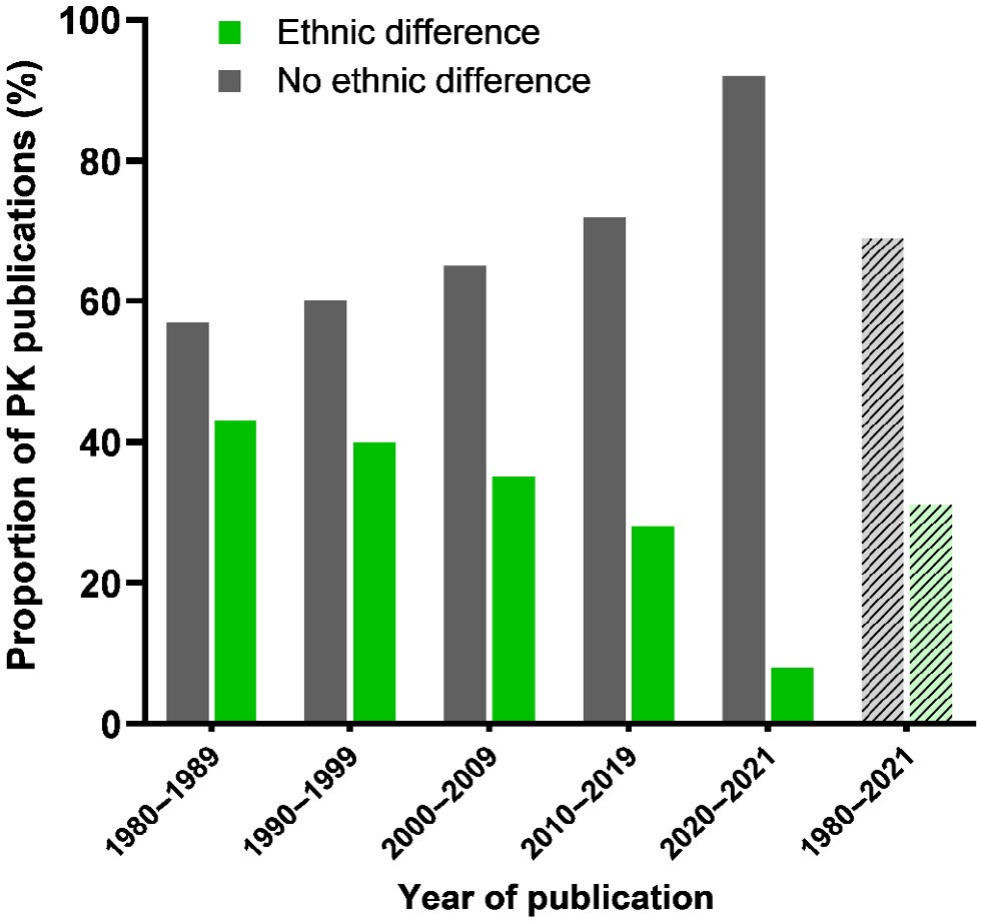
Comparison between decades of published data which showed significant pharmacokinetic differences between ethnic groups (green) and which showed no significant pharmacokinetic differences (gray). Studies identified in 2021 was between January and March. Two hundred and thirty‐seven studies were compared which included 163 studies that showed no significant difference in pharmacokinetics and 74 which showed significant difference in pharmacokinetics

Overall, of the research articles, the majority (69%) did not find significant ethnic differences in pharmacokinetic processes (Figure 2). From 1980 to 2019, there was an increase in numbers of papers investigating ethnic differences in PK (14 papers in 1980–1989; 33 papers in 1990–1999; 54 papers in 2000–2009; and 124 papers in 2010–2019) and there was an increase in the proportion of papers reporting no significant difference with a corresponding decrease in the proportion reporting significant difference (Figure 2). Papers which showed no statistically significant ethnic differences can be found in Supplementary Material 2, Table S3.

### 3.4 Summary of responses from the respondents

Table 2 summarizes the current (or past) teaching practice of academics in HE relating to ethnic differences in PK. There were 21 questionnaire respondents: 13 from the United Kingdom with 8 from a range of other countries. All except one respondent were from Higher Education Institutions with one from industry. Though all respondents listed one or more ADME processes which are likely to show differences in ethnic diversity, five had not previously or currently taught inter‐ethnic differences in PK. Ninety‐five percent of respondents thought metabolism was likely to show inter‐ethnic differences while less than 30% of respondents thought that absorption, distribution, or excretion were likely to show inter‐ethnic differences in PK. A complete breakdown of anonymized responses from respondents is detailed in Supplementary Material 2, Table S2.

**TABLE 2:** Respondents’ responses to questionnaire related to inter‐ethnic differences in ADME

	Absorption	Distribution	Metabolism	Excretion
Pharmacokinetics processes currently or previously taught by the respondents	18	18	20	20
Respondents who think ADME most likely to show inter‐ethnic differences	3	4	20	6

Of the 15 respondents who taught inter‐ethnic differences in PK, only one discussed examples that were not necessarily related to drug metabolism. This respondent stated, “Without detailing the processes, racial differences affect transporters (absorption, metabolism, and excretion), enzymes (metabolism) and body composition (distribution and possibly metabolism and excretion).” Notably, of all the respondents who taught inter‐ethnic differences in PK, 80% attributed genetic polymorphisms to inter‐ethnic differences in PK.

### 3.5 Summary of PK processes associated with inter‐ethnic difference in PK from published literature and questionnaires

**FIGURE 3:**
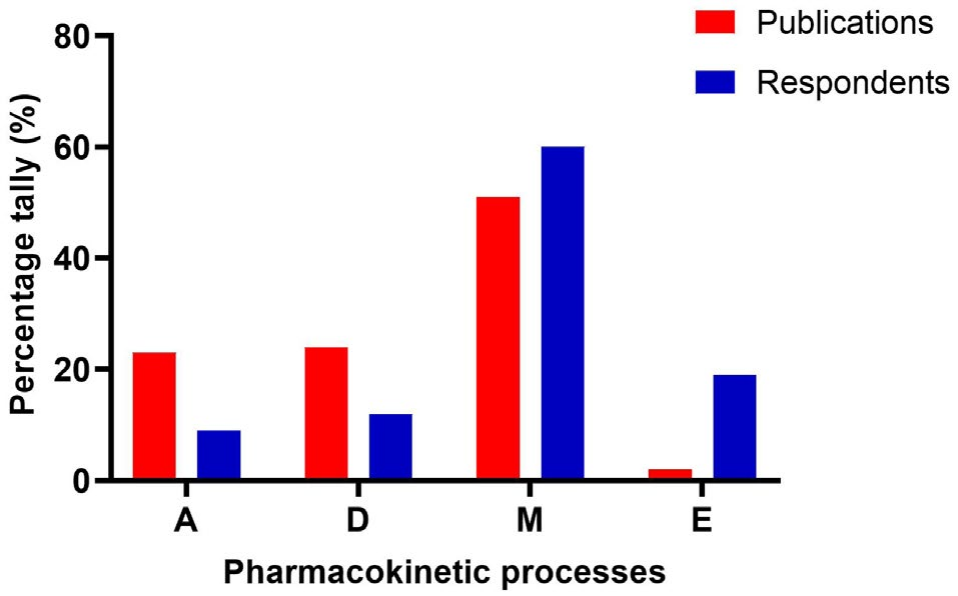
Comparison of current teaching and the literature publications for each pharmacokinetic process. Literature percentage tally was based on the number of papers that linked PK differences to the ADME processes in comparison to the total number or papers. Respondent percentage tally was based on the numbers of respondents who thought there were ethnic differences in one or more of the ADME processes compared to the total number or respondents. The percentage tallies for literature evidence and respondents were weighed equally. Absorption: A, Distribution: D, Metabolism: M, and Excretion: E

In both the literature and current HE teaching, the process of metabolism appears to be the most reported mechanism linked to ethnic differences making up 60% of taught material and 50% of reported literature (Figure 3). Our literature search identified higher frequencies of papers suggesting inter‐ethnic differences in absorption and distribution compared to the respondents’ answers. Absorption and distribution made up 8%–12% of teaching respondents compared to about 23% in the reported studies analyzed (Figure 3). On the other hand, our literature search had lower frequencies of papers reporting inter‐ethnic differences linked to excretion (2%) compared to the respondents’ (19%).

### 3.6 Suggested or investigated mechanisms linked to inter‐ethnic PK differences in published literature

Genetic polymorphism was the predominant mechanism (37%) proposed or suggested by authors as an explanation for the inter‐ethnic difference in PK observed in their studies. Other nongenetic explanations that were proposed included diet, environmental/lifestyle factors such as alcohol consumption and tobacco intake, physiological factors such as body weight, biochemical factors such as expression levels of plasma proteins, transporters, and enzymes. Inter‐ethnic difference in enzyme activity was also proposed as a probable mechanism.

## 4 DISCUSSION

Several factors can contribute to interindividual variation in PK. Some of the factors might be more common in certain ethnic groups than others. It is important to understand what physiological, socio‐cultural factors, and other underlying mechanism may lead to interindividual differences in PK. It is equally important that a proper reflection of how these factors could impact on interindividual differences in PK is included as a part of PK‐related teaching in HE. This study aimed to review evidence from literature which suggest ethnic differences in PK, identify the mechanism proposed by the authors, investigate how current teaching addresses these factors, and make recommendations for teaching inter‐ethnic differences in PK in HE.

### 4.1 Terminology and methodology

One major observation from this review is the use of the terms “ethnicity” and “race” which are often used interchangeably and without definition although “ethnicity” is often used to indicate more social and cultural characteristics. The current United Nations recommendation is that individuals are asked to self‐report their ethnicity rather than it being assumed by the investigator^4^ however the majority of the papers included in this review do not specify how participants were characterized in their respective groups. The way that ethnic groups are defined varies around the world: one paper referred to an ethnic group as “Hispanic” however this is open to interpretation as individuals identifying as such may have ancestry of European, Native American, or African descent.^22^ While we have searched for literature citing ethnic differences, the implied meaning of the term in these contexts is generally closer to that of race since many papers frequently used the terms “Caucasians,” “Whites,” or “Blacks” which refer to large groups of peoples each of which have diverse social and cultural backgrounds. It is important to note that some authors used terminology which refined ethnic groups further by specifying “American‐born Asians” to “Asian‐born Asians” or sub‐populations in China (Koreans, Hans, Uighurs, Mongolians, and Huis).^23^

Many of the studies report quite small sample sizes including typically between 8 and 30 individuals for each ethnic group which raises the question of whether such a small sample size can possibly be representative of a particular ethnic group. For example, Zhou et al., (1990) reported statistically significant differences in propranolol plasma protein binding in a study of 8 Chinese and 8 White men^16^ while Sowinski et al., (1996) reported statistically significant differences in clearance of propranolol in a study of 12 White and 13 Black males.^24^ In both cases, statistical significance was reached using a one‐tailed *t*‐test however, a two‐tailed test may not have reached statistical significance. It is a matter of debate as to which statistical test is more appropriate.

Small sample size also leads to questions about whether sampling is effective. In a study of five men and five women of five different ethnicities within China (Han, Mongolian, Korean, Hui, and Uighur) differences in half‐life were seen between Han and Mongolian subjects but not between the other ethnic groups.^25^ However, in other studies where a Chinese group was compared with Whites as reported in Zhou et al., (1990) and Chu et al., (2014) reporting 8 and 14 Chinese, respectively,^16^, ^26^ it was not clear how the individuals were chosen and which Chinese ethnic group(s) they came from. In other studies, the pharmacokinetic parameters of one ethnic group were compared with those in the published literature, for example, the PK of felodipine in 30 Mexicans^27^ and the PK of levosimendan in 14 Chinese males^26^ were compared to reported PK of respective drug obtained from published literature with little attempt to account for potentially confounding variables leading to questions about whether the difference was genuinely to do with ethnicity or some other factor(s) that distinguished the two groups. These studies could provide a rich source of material for students to critique experimental design and statistical analysis.

### 4.2 Summary of published literature identified in the review

#### 4.2.1 Number of papers reporting similarities in and differences between ethnic groups increase each decade

Only thirty‐one percent of the research articles identified in the current study reported difference in the PK of drugs between ethnic groups (Figure 2), suggesting that, in understanding variabilities in the PK of drugs between ethnicities, there is perhaps more that unites than divides. Through the decades, there is an increasing number of papers investigating ethnic differences in the PK of drugs which mirrors the increase in the size of the scientific literature. Interestingly, there is a decreasing proportion of papers finding a significant difference between ethnic groups (Figure 2). One can only speculate why this decrease has occurred: perhaps it is because it is becoming more acceptable to publish statistically nonsignificant results or perhaps there is decreasing expectation that ethnic differences have a biological basis. It is interesting to note that, generally, the older papers used in this study frequently used terms such as “Blacks” and “Whites,” whereas the more recent papers used terms such as “American‐ born Asians,” distinguishing between types of Asians such as East Asians and distinguishing between Blacks such as African Americas, Ghanaians, and Ugandans. This might reflect changing perspectives on the biological basis for racial and ethnic categories.

#### 4.2.2 Metabolism and genetic polymorphism are frequently attributed to inter‐ethnic difference in PK in published literature and in current PK teaching

**FIGURE 4:**
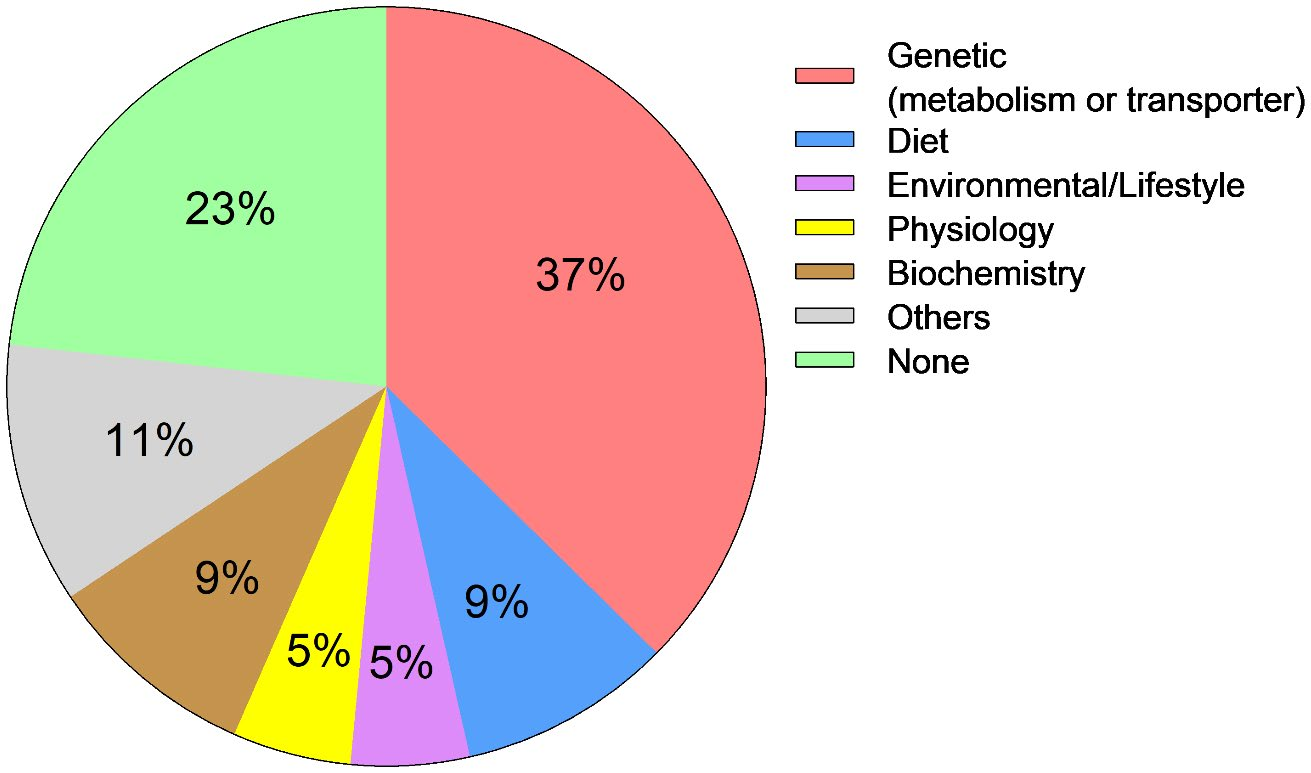
Suggested or proposed mechanisms linked to inter‐ethnic PK differences in published literature. Suggested explanations for inter‐ethnic difference in PK reported by authors of published literature included in this study. Genetic: genetic polymorphism in transporters or metabolism enzymes; diet: food consumption in participants; environmental/lifestyle: alcohol intake and/or smoking tobacco; physiology: weight and blood flow; biochemistry: enzyme or transporter expression levels, plasma protein levels; others: differences in enzyme activity; none: no general explanation or author provide no link between study observation and one particular ethnicity

In published literature and the current teaching practice, most ethnic sensitivity to PK is thought to involve drug metabolism (Figure 3; Tables 1 and 2), largely relating to genetic polymorphisms of metabolizing enzymes (Figure 4). Only one respondent reported teaching examples that reflected other nongenetic reasons. It can be argued that a reason for the high representation of metabolism (more precisely, genetic polymorphisms), is that this process drives the systemic availability and variability of a drug concentration associated with therapeutic levels of a drug and ensuring right dosing strategies that will avoid supratherapeutic or subtherapeutic systemic concentration across a wide range of individual.^28^

### 4.3 Mechanisms underlying interindividual differences in pharmacokinetics

#### 4.3.1 Diet and its effect on drug absorption, distribution, and metabolism

Diet can change drug disposition by altering the luminal pH, direct binding to drug, gastric emptying, intestinal transit, mucosal absorption, hepatic blood flow, and induction of enzymes.^5^ It has also been shown that for certain lipophilic antimalarials like lumefantrine, there is 16‐fold increase in bioavailability if the drug is administered with a high‐fat meal.^29^ This is thought to be because the consumption of fatty meals increases in vivo solubility of lumefantrine as a result of increased bile micelles production.^30^ In this present study, many papers reported diet to be a crucial factor responsible for some inter‐ethnic differences in PK. For example, both modafinil^31^ and ziprasidone^32^ were investigated in the five prominent ethnic groups in China and diet was implicated for the pharmacokinetic differences observed (Table 1). It was thought that this may be due to the predominantly fat and protein diet eaten by the Mongolians compared to the agriculturally based diet of the Hans.^32^ Moreover, absence/presence of meat‐based diet was implicated in paracetamol distribution between subjects from Hong Kong, Pakistan, Denmark, Spain, and South Africa^33^ (Table 1), and for the absorption of alisertib in West & East Asian and Western subjects^34^ (Table 1). In our survey of teaching practices, when asked to provide examples of taught inter‐ethnic pharmacokinetic differences in HE, diet was not discussed. This may provide an explanation as to why there was a lower proportion of absorption and distribution (8% and 12%, respectively) reported in the current teaching compared to literature (23% for both processes) (Figure 3). Of all the studies identifying inter‐ethnic differences in PK, 9% suggested diet as possible reason for the inter‐ethnic difference observed (Figure 4).

#### 4.3.2 Body weight as a physiological factor and its effect on distribution

The distribution of a drug has many determinants including tissue binding, organ blood flow, and drug plasma protein binding.^35^ Furthermore, in obese patients, tissue blood flow is decreased, and cardiac function and structure are modified.^35^ In Asian populations, the average body weight is 60 kg^36^ which is significantly lower than many other populations. This is reflected with the drugs efavirenz^36^ and selumetinib^37^ where body weight was postulated as the underlying reason for the observed distribution differences seen between the studied populations (Table 1). Furthermore, lower body weight has also been considered a risk factor for increased systemic exposure of certain drugs such as tenofovir disoproxil fumarate (TDF) which may therefore provide justification for adjusting dosing regimens.^36^ Notably, no respondent mentioned inter‐ethnic differences in weight as an example of what they would teach their students on inter‐ethnic differences in PK.

#### 4.3.3 Metabolizing enzyme and transporter abundance and polymorphism and effects on drug absorption, distribution, and metabolism

Genetic polymorphisms of drug‐metabolizing enzymes have been widely reported. There were several references made to the prevalence of CYP P450 enzyme polymorphisms in different ethnic groups noted in this review (Table 1). This is in agreement with what is generally known that these polymorphisms show varying frequencies in different ethnic groups, that is, that there can be differences in prevalence but no polymorphism is unique to a particular ethnic group. For example, there are higher frequencies of CYP3A5 allele expression (CYP3A5*3, CYP3A5*6, and CYP3A5*7) in African Americans compared to European, Native Americans, and Asian ancestry^38^ and higher expression of the slow metabolizer of EFV CYP2B6516 G > T allele in Africans Americans (46.7%) and Sub‐Saharan Africans (45%) compared to Asians (17.4%), Hispanics (17.4%), Japanese (18%), and Caucasian (21.4%).^21^ Also, in the metabolism of morphine and debrisoquine, the frequency of poor metabolizer allele of CYP 2D6‐dependant oxidation polymorphisms is higher in the Caucasians (6%–10%) compared to the Chinese (0%–0.7%) (Table 1).^39^ This means that, in practice, if it is important to know whether a patient is a fast or slow metabolizer then a test to determine that should be performed, rather than using ethnicity as a proxy. Indeed, it has been proposed that the use of race as a proxy for biological variables should be avoided in medicine.^40^

In addition to isoforms of metabolizing enzymes, there were also references to genetic polymorphism or protein abundances relating to plasma proteins particularly α_1_ acidic glycoprotein (AAG). For example, the level of AAG‐bound propranolol was significantly lower in the Chinese compared to Caucasians (Table 1).^41^ Genetic polymorphisms of membrane‐bound efflux transporters such as P‐gp may influence absorption and is one of the proposed mechanisms for anticancer drug resistance.^42^ Another example of polymorphism occurs in organic anion‐transporting polypeptide (OATP) transporter superfamily. Located on the basolateral membrane of hepatocytes, these proteins are involved in the uptake and clearance of many drugs therefore influencing absorption and excretion.^43^ It has been reported that there may be differences in the PK of transporter substrates between Caucasians and Asians.^44^ Interestingly, with the same genotype, OATP1B1, the activity in an Asian population is still half that of Caucasians suggesting that besides allele frequencies of transporter genes, there is an underlying ethnic variability, distinct from genetics, that contributes to the pharmacokinetic variations.^44^ In the survey of teaching practices, respondents mainly provided examples related to genetic polymorphism of CYPs and OATP1B1 as what they would teach students on the subject of inter‐ethnic variabilities in drug disposition (see Supplementary Material Table S2). Genetic polymorphism was the predominant mechanism suggested in the literature identified in the review as linked to inter‐ethnic differences in drug disposition (Figure 4).

The importance of polymorphisms of metabolizing enzymes and transporters as a mechanism associated with inter‐ethnic differences in PK has received much greater attention in the literature (Table 2 and Figure 4) and in HE in PK (Table 2; Figure 3) although it remains to be seen whether this increased attention reflects the importance of this mechanism or whether it is the result of researchers and teachers selecting this for their attention. It is important to note that the attribution of inter‐ethnic differences in PK to genetic polymorphism that some authors in this review made were postulated, and their actual studies neither confirmed nor refuted their postulation. This may, therefore, increase the possibility of unconscious bias when interpreting these studies and may lead to attributing nearly all unexplained variabilities in PK to genetic polymorphisms. It is important to note that, while there is genetic diversity between individuals, different races or ethnicities cannot be categorized on the basis of their genetics as greater genetic diversity has been reported within an ethnic groups compared to between ethnic groups, for example, there is greater genetic diversity within Africans than between Africans and other non‐African populations.^3^, ^45^

### 4.4 What does this all mean for teaching PK in the future?

We recommend that educators explain the variety of factors that can lead to interindividual variation in PK rather than focussing on inter‐ethnic differences. These factors include diet, weight, age, concurrent medicines, co‐morbidities, isoforms of metabolizing enzymes and transporters, differing levels of expression of plasma proteins, enzymes, and transporters, and use a balanced array of examples that include both environmental and genetic factors.

The evidence upon which current PK teaching discusses variabilities in the PK of drugs between ethnicities as being predominantly linked to genetic polymorphism is at best weak. No genetic trait is unique to one race or ethnicity^46^ and traits are influenced by both environmental and genetic factors. Educators should ensure that students recognize that there is greater genetic diversity within ethnic groups than between them and should reinforce the consensus view among geneticists that there is no biological or genetic basis to the idea of race.^3^ Therefore, single‐nucleotide polymorphisms should be discussed primarily in the context of interindividual variation in PK, rather than inter‐ethnic differences in PK.

While evidence for varying prevalence of certain SNPs among ethnic groups could be discussed, this should be in the context of a critical analysis of the prevalence of the resultant PK variabilities within the ethnic group and the potential pitfalls in using race or ethnicity as a proxy for a variable(s) that could be measured directly. This analytical perspective provides scope for discussions around the rationale behind ethnic‐dependent use or dosing of certain medicines in current teaching^40^ for example, there is considerable debate surrounding the perceived genetic interpretation of race‐dependent dose–response relationship associated with the use of angiotensin‐converting enzyme inhibitors (ACEIs) among Africans or African Americans. Other mechanisms may explain variability in response to ACEIs observed in African American and “Whites,” for example Flack et al., (2000) suggested that differences observed in response to low‐dose ACEI among African American may in fact be due to relatively higher dietary sodium intake common among African Americans.^47^

A limitation of this study was that while assessing the literature to determine what pharmacokinetic parameters were affected, the methodology or the results of only some of the papers used were scrutinized. As we have mentioned, this is important because the number of participants for some studies were low, and this raises the question of whether this provides enough information that could be extrapolated to an entire ethnic group. Further work is required to systematically review drug classes and critically examine the methodology, terminology, and prior assumptions. It is important to note that when filling out the questionnaire, many respondents focused more on general examples when teaching, such as overviews of polymorphisms of CYP450 enzymes or patterns observed, whereas the literature looks at more specific examples. In this review, papers identified were classified by pharmacokinetic (absorption, distribution, metabolism, or excretion) differences based on the discussion of the paper or may have been inferred from the experimental findings rather than being directly studied in the article. Also, the search terminologies utilized may have limited our ability to identify all published literature where the effect of inter‐ethnic differences in the PK of drugs was studied, as some papers in which ethnic differences in PK has been observed were not included in the list of identified papers using the search terms employed in this current study, for example, the studies reported by Li et al., (2007) and Tanii et al., (2011).^48^, ^49^ The interpretation of educators’ perspective about inter‐ethnic differences in PK in this present study was based on a small size and limited geographical representation of respondents, therefore, an extrapolation of our findings on general educators’ teaching practices would be difficult to reach. Another limitation of this review was that no qualitative research methodologies were used to analyze the narrative text response from the respondents.

## 5 CONCLUSION

There is a considerable literature comparing PK between ethnic groups with more papers reporting similarities than differences and a decrease in the proportion of papers showing ethnic differences over time. Much of this literature focusses on polymorphisms in mobilizing enzymes and transporters and this is reflected in HE teaching. This overemphasis on genetic mechanisms results in less emphasis on other factors that contribute to interindividual variation in PK including diet, concurrent medicines, weight, age, illness, and varying levels of plasma proteins, metabolizing enzymes, and transporters.

Logically though, if we know, for example, that weight can have an impact on PK, then it would be better to assess a patient's weight when prescribing rather than using ethnicity as a proxy. The same is true for the other factors that can lead to interindividual variation.

In our analysis, we identified some methodological issues with some of the studies that report differences in PK between ethnic groups. One aspect of this was in the definitions of the terms used to describe ethnicity and a lack of clarity on how the term “ethnicity” was determined. Another aspect is in the experimental design, sampling, and statistical analysis in some of the papers and it will be important to pursue this further by focussing on certain classes of drugs to weigh up the strength of the evidence for and against ethnic differences. This information will provide a rich source of material in teaching of critical thinking.

## CONFLICT OF INTEREST

The authors declare no conflict of interest.

## AUTHORS CONTRIBUTIONS

OO, NK, and JF contributed to the conception and design of the research and the acquisition, analysis and interpretation of data generated. OO, NK, JW, and JK were all involved in drafting the manuscript and revising it for important intellectual content.

## Supporting information

Supplementary Material

## Data Availability

The data that support the findings of this study are available from the corresponding author upon reasonable request.
