# Supplementary material for: Inter‐ethnic differences in pharmacokinetics—is there more that unites than divides?
Source: Pharmacol Res Perspect. 2021 Nov 2;9(6):e00890. doi: 10.1002/prp2.890 (PMC8561230; doi:10.1002/prp2.890)

**Supplementary material**

**Table S1.** Search terms used from the PubMed and SCOPUS database.

| PubMed | (((((((ethnic* difference* in pharmacokinetics) OR (ethnic* pharmacokinetic* difference* drug absorption)) OR (ethnic* pharmacokinetic* difference* drug distribution)) OR (ethnic* pharmacokinetic* difference* drug metabolism[Title/Abstract])) OR (ethnic* pharmacokinetic* difference* drug excretion[Title/Abstract])) OR (ethnic* pharmacokinetic* difference* physiology[Title/Abstract])) OR (ethnic* pharmacokinetic* difference* biochemistry[Title/Abstract])) OR (ethnic* pharmacokinetic* difference* drug disposition[Title/Abstract] |
| --- | --- |
| SCOPUS | ( TITLE-ABS-KEY ( ethnic* )  AND  TITLE-ABS-KEY ( differences )  AND  TITLE-ABS-KEY ( pharmacokinetics )  AND  TITLE-ABS-KEY ( drugs )  OR  TITLE-ABS-KEY ( administration )  OR  TITLE-ABS-KEY ( distribution )  OR  TITLE-ABS-KEY ( metabolism )  OR  TITLE-ABS-KEY ( excretion )  OR  TITLE-ABS-KEY ( african  AND american )  OR  TITLE-ABS-KEY ( blacks )  OR  TITLE-ABS-KEY ( asian )  OR  TITLE-ABS-KEY ( caucasian )  OR  TITLE-ABS-KEY ( european )  OR  TITLE-ABS-KEY ( biochemistry )  OR  TITLE-ABS-KEY ( physiology )  OR  TITLE-ABS-KEY ( disposition )  AND NOT  TITLE-ABS-KEY ( sex )  AND NOT  TITLE-ABS-KEY ( gender )  AND NOT  TITLE-ABS-KEY ( age )  OR  TITLE-ABS-KEY ( absorption ) )  AND  ( LIMIT-TO ( LANGUAGE ,  "English" ) ) |

**Figure S1.** Questionnaire set up on social media platforms (Twitter, LinkedIn, The University of Nottingham website, and the British Pharmacological Society).


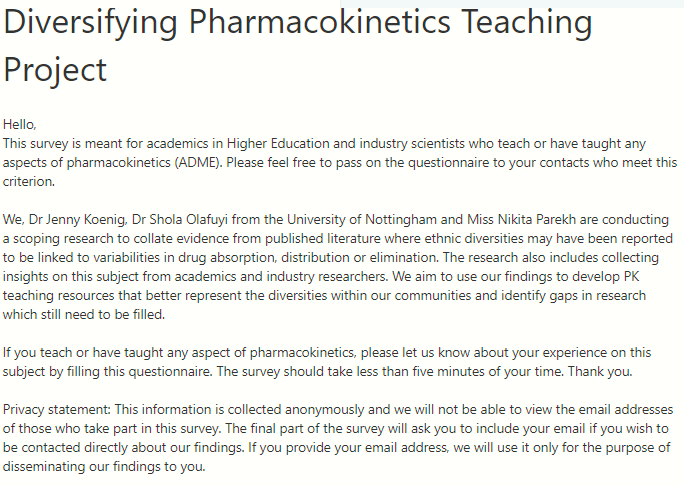


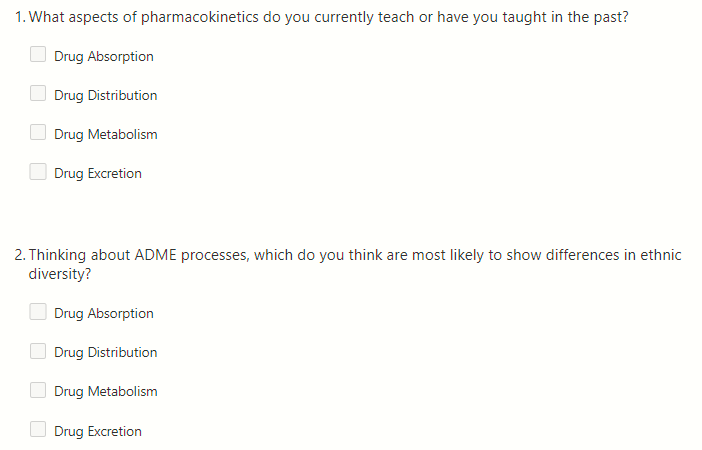


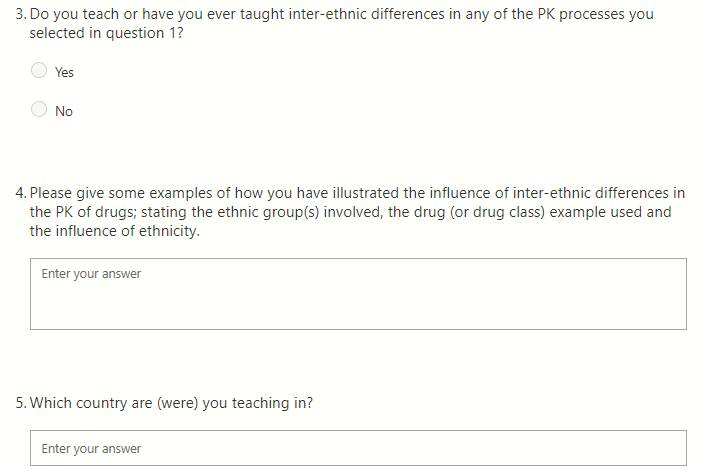


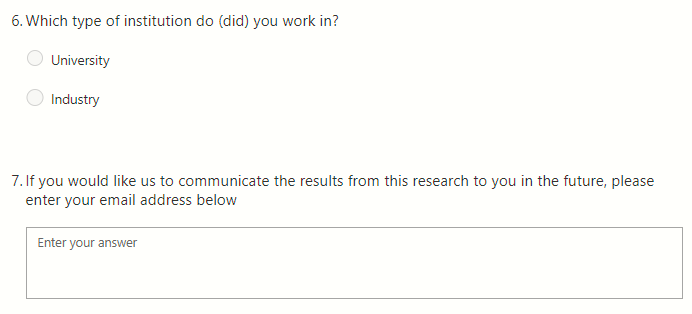

Supplement: Supplementary file 1 — Supplementary Material [file PRP2-9-e00890-s001.zip › prp2890-sup-0001-SupInfo.docx]
